# The impact of national prenatal screening on the time of diagnosis and outcome of pregnancies affected with common trisomies, a cohort study in the Northern Netherlands

**DOI:** 10.1186/s12884-016-1203-6

**Published:** 2017-01-05

**Authors:** Katelijne Bouman, Marian K. Bakker, Erwin Birnie, Lies ter Beek, Caterina M. Bilardo, Irene M. van Langen, Hermien E. K. de Walle

**Affiliations:** 1 University of Groningen, University Medical Center Groningen, Department of Genetics, PO Box 30001, 9700 RB Groningen, The Netherlands; 2 University of Groningen, University Medical Center Groningen, Department of Obstetrics, PO Box 30001, 9700 RB Groningen, The Netherlands

**Keywords:** Prenatal screening, Trisomy 21, Trisomy 13, Trisomy 18, Maternal age, Pregnancy outcome

## Abstract

**Background:**

To evaluate the impact of the introduction of prenatal screening on time of detection and pregnancy outcome for trisomy 21 (T21), trisomy 18 (T18) and trisomy 13 (T13).

**Methods:**

We performed a retrospective, population-based cohort study in the Northern Netherlands including 503 trisomy cases born between 2005 and 2012. Screening tests and invasive procedures, timing of diagnosis and pregnancy outcome were compared between the period before (2005–2006) and after introduction (2007–2012) using *X* ^2^ tests.

**Results:**

There was an increase in proportion of women who had a prenatal screening and/or invasive test, from 62% in 2005–2006 to 84% in 2010–2012 (*p* < 0.01), while the proportion of prenatally diagnosed cases did not change (60% overall). In women < =35 years 47% of the cases were diagnosed prenatally vs 73% in women >35 years (*p* < 0.01). More T13/T18 cases were diagnosed <24 weeks after introduction (62% vs 84%; *p* < 0.01). In T13/T18 intra-uterine death decreased (26% vs 15%), while terminations increased: 55% vs 72%.

**Conclusion:**

The introduction of prenatal screening had limited impact on the time of detection and outcome of the most common trisomies. The introduction of the 20-week anomaly scan has resulted in more trisomy cases diagnosed <24 weeks and a shift from fetal death to terminations.

## Background

The Netherlands have an exceptional position in Europe when it concerns prenatal screening (PNS). Before 2007, PNS for the common trisomies -trisomy 21 (T21), trisomy 18 (T18) and trisomy 13 (T13)- was based on advanced maternal age of over 35 years (AMA). First and second trimester PNS based on serum markers and/or nuchal translucency (NT) were available, but only on request of the pregnant woman [1]. A structural anomaly scan (SAS) at 20 weeks gestation aimed at screening for congenital anomalies was not available nationwide. Scans were only performed for dating and medical reasons e.g., suspicion of intrauterine growth restriction or excessive growth or a positive family history for fetal anomalies.

Since January 2007, a nationwide prenatal screening program has been implemented in the Dutch health care system, in which the combined test (CT) in the first trimester to determine the risk for T21 and a SAS for neural tube defects in the second trimester are offered to all pregnant women. In 2010 the CT expanded to determine also the risk for T18 and T13. PNS is offered after counselling and women are free to refrain from PNS. The Dutch Association of Obstetricians and Gynaecologists decided that not only the spine, but the entire fetus should be examined. Ultrasound quality and knowledge of the association between congenital anomalies or softmarkers and common trisomies has rapidly increased enabling the use of SAS as an additional way of PNS for common trisomies. Women over 35 years could also opt for direct prenatal diagnosis through an amniocentesis or chorion villous sampling. The costs of the CT were reimbursed for this age category, whereas the younger women had to pay for the CT themselves (approximately 160 Euros). The SAS is reimbursed for all pregnant women.

Since the introduction of PNS for congenital anomalies in other West European countries, more pregnancies are terminated and fewer babies with common trisomies are born alive in these countries [2–6]. A recent study from Western Australia also found a significant reduction in the rate of liveborns with Down syndrome [7]. The aim of this study was to evaluate the impact of the implementation of PNS in the Northern Netherlands on the detection of common trisomies: T21, T18 and T13 with respect to both first and second trimester PNS, time of diagnosis and pregnancy outcome. In addition we compared the impact of the implementation of PNS on pregnancy outcome for type of trisomy (T21 vs T13/T18) and for women in two different age categories (<=35 years and >35 years).

## Methods

We performed a retrospective cohort study on cases diagnosed with T21, T18 or T13, with a date of delivery or termination of pregnancy (TOP) between 1-1-2005 and 31-12-2012 and for which the mother lived in one of the three Northern provinces at the time of birth.

Cases diagnosed prenatally or postnatally with T21, T18 or T13 were identified from the database of the cytogenetic laboratory for three tertiary feto-maternal units for prenatal diagnosis held at the University Medical Centre of Groningen (UMCG). The cytogenetic laboratory of the UMCG performs all pre- and postnatal karyotyping in the Northern Netherlands. Standard trisomies, Robertsonian translocations leading to a full T21, T18 or T13, T21, T18 or T13 mosaicisms and a diagnosis of T21, T18 or T13 by quantitative fluorescence-polymerase chain reaction (QF-PCR) or multiplex ligation-dependent probe amplification (MLPA) in case of culture failure were included. Partial T21, T18 and T13 due to a reciprocal translocation were excluded. The database of the cytogenetic laboratory contains all genotyped cases of T21, T18 or T13 diagnosed in the three Northern provinces. Therefore the identification of cases with these common trisomies can be considered complete.

The first cohort contains cases born in 2005 and 2006, from mothers that were not offered PNS on a routine base. The second cohort contains cases born between 2007 and 2012, with a gestational age of at least 10 weeks at January 1st 2007, from mothers that were offered PNS routinely as part of the nationwide screening program. The information on PNS, prenatal diagnosis and pregnancy outcome was retrieved from matching data from the regional prenatal screening database, the prenatal ultrasound database from the fetal medicine unit of the UMCG and the regional congenital anomaly registry EUROCAT Northern Netherlands.

### Prenatal screening

For each case included in the study population, we recorded whether any form of PNS was performed regardless of the trimester and independently of the outcome of PNS.

Prenatal screening in the first trimester (PNS1) was defined as performed, if a risk assessment for T21 was carried out based on more than maternal age only. In the 2005–2006 cohort, risk assessment was occasionally performed on the basis of either serum markers or nuchal translucency. We defined these cases as “first trimester PNS performed”, as the aim of the test was to estimate the risk for T21. In the 2007–2012 cohort PNS1 was defined as the combined risk assessment for T21 based on serum free beta human chorionic gonatropin (fβ HCG), serum pregnancy associated plasma protein A (PAPP-A), an NT measurement and maternal age using the formulas of the fetal medicine foundation. A high proportion of the fetuses with T18 and T13 is detected with the T21 algoritm as well [8–10].

Second trimester PNS (PNS2) was defined as performed if a second trimester SAS or a second trimester serum PNS with serum markers HCG and AFP (alfa fetoprotein) was accomplished. The aim of PNS by SAS is to determine in an uncomplicated pregnancy the presence of congenital anomalies or softmarkers, and therefore can also be considered as a screening tool for common trisomies.

Ultrasounds performed on parents request and without medical indication and ultrasound scans performed for dating or on medical indications (a priori risks, pathologies suspected in the pregnancy etc.) were not considered as PNS. Therefore, anomalies diagnosed “accidentally” in these cases and followed by prenatal diagnosis (PND), were considered diagnosis outside the PNS program. AMA as such was not defined as a first nor as a second trimester PNS tool for T21, T18 or T13.

### Prenatal diagnosis

PND was defined as (molecular) karyotyping after chorionic villus sampling (CVS) or amniocentesis (AC), performed by licensed feto-maternal specialists in one of the three tertiary feto-maternal units in the Northern Netherlands. A CVS or an AC could be performed following first or second PNS, in mothers of AMA, or in pregnancies in which anomalies are detected on ultrasound for medical indication. Karyotyping performed after fetal death was not considered as PND.

### First prenatal test performed

For each case we determined which test was performed first in the pregnancy: PNS1, PNS2 or PND. If PNS1 was followed by PND, PNS1 was recorded as the first prenatal test performed, if PND was performed without PNS1 or PNS2, PND was recorded as first prenatal test and so on. Cases with a postnatal diagnosis and no information on PNS or PND were classified as no ‘PNS or PND performed’.

### Time of diagnosis

The time of diagnosis was recorded as the time the (molecular) cytogenetic diagnosis was made: prenatally < 24 weeks, prenatally > = 24 weeks or postnatally. Cases with specific ultrasound anomalies suspect for a trisomy but without prenatal karyotyping and cases with a post mortem cytogenetic diagnosis were defined as postnatally diagnosed.

### Pregnancy outcome

Pregnancy outcome was defined as fetal death (miscarriage and intrauterine fetal deaths after 24 weeks gestation), TOP or live birth. Live born cases, which died after birth, were defined as live births.

### Data analysis

We compared the first prenatal tests performed, time of diagnosis and pregnancy outcome before (2005–2006) and after implementation of the PNS (2007–2012). For certain analyses we divided the period after implementation in 2 sub periods (2007–2009 and 2010–2012) to investigate if a trend was present. We also stratified the analyses between type of trisomy (T21 vs T13/T18) and between two age categories (<=35 years and >35 years). T13 and T18 were combined in one group as these trisomies have a comparable presentation in pregnancy, with respect to the first trimester risk assessment with the T21 algorithm [10, 11].

We compared the proportion of cases who received PNS1, PNS2 and PND as the first test in pregnancy between the time periods, but we also compared the cumulative proportion of cases taking these different types of tests: PNS1, PNS1 and/or PNS2, PNS1 and/or PNS2 and/or PND between the time periods and stratified for maternal age.

The data were analysed by IBM SPSS Statistics (version 20). Chi square (*X* ^2^) or Fisher Exact Test was used to investigate whether the proportions were statistically different between the period before and after implementation. A *p*-value < 0.05 (two sided) was considered a statistically significant difference.

## Results

The study population consisted of 503 cases: 331 (66%) T21, 135 (27%) T18 and 37 (7%) T13. For 53% of the cases the mothers were over 35 years (7 cases with missing information on maternal age). During the study period, 141,789 children were born in the Northern Netherlands. The total prevalence of T21 was 23.3 per 10,000 births; 13.2 per 10,000 births in women < = 35 years and 79.4 per 10,000 birth in women >35 years.

In Table 1 results are presented on first prenatal test performed, time of diagnosis and pregnancy outcome for the study population, according to year of birth and further specified according to maternal age (Table 2) and type of common trisomies (Table 3).

**Table 1:** First prenatal test, time of diagnosis and pregnancy outcome according to year of birth in cases with a common trisomy (T21, T18, T13)

	Year of birth							
	2005–2006		2007–2009		2010–2012		Total	
First prenatal test*								
PNS1	47	37%	72	39%	87	46%	206	41%
PNS2	4	3%	52	28%	57	30%	113	22%
PND	29	23%	23	12%	14	7%	66	13%
No PNS or PND	48	38%	40	21%	30	16%	118	23%
Time of diagnosis**								
Postnatally	52	41%	79	42%	71	38%	202	40%
Prenatally < 24 weeks	64	50%	104	56%	108	57%	276	55%
Prenatally > = 24 weeks	12	9%	4	2%	9	5%	25	5%
Pregnancy outcome***								
Live birth	55	43%	79	42%	76	40%	210	42%
Fetal death	14	11%	15	8%	24	13%	53	11%
TOP	59	46%	93	50%	88	47%	240	48%

*T21* trisomy 21, *T18* trisomy 18, *T13* trisomy 13, *PNS1* prenatal screening in first trimester, *PNS2* prenatal screening in second trimester, *PND* prenatal diagnosis, *TOP* termination of pregnancy

*X ^2^ = 60.2, *p* < 0.001; *X*2 for trend = 23.5, *p* < 0.01

**X ^2^ = 9.3, *p* = 0.054

***X ^2^ = 2.4, *p* = 0.658

**Table 2:** First prenatal test, time of diagnosis and pregnancy outcome according to year of birth in cases with a common trisomy (T21, T18, T13) in women < =35 years and >35 years

	Year of birth					
	2005–2006		2007–2012		Total	
Women < = 35 years	*N* = 61		*N* = 169		*N* = 230	
First prenatal test**						
PNS1	15	25%	42	25%	57	25%
PNS2	1	2%	76	45%	77	33%
PND	12	20%	9	5%	21	9%
No PNS or PND	33	54%	42	25%	75	33%
Time of diagnosis**						
Postnatal	34	56%	89	53%	123	53%
Prenatal < 24 weeks	18	30%	73	43%	91	40%
Prenatal > = 24 weeks	9	15%	7	4%	16	7%
Pregnancy outcome						
Live birth	34	56%	94	56%	128	56%
Fetal death	8	13%	17	10%	25	11%
TOP	19	31%	58	34%	77	33%
Women >35 years*	*N* = 67		*N* = 199		*N* = 266	
First prenatal test**						
PNS1	32	48%	116	58%	148	56%
PNS2	3	4%	33	17%	36	14%
PND	17	25%	28	14%	45	17%
No PNS or PND	15	22%	22	11%	37	14%
Time of diagnosis						
Postnatal	18	27%	54	27%	72	27%
Prenatal < 24 weeks	46	69%	139	70%	185	70%
Prenatal > = 24 weeks	3	4%	6	3%	9	3%
Pregnancy outcome						
Live birth	21	31%	54	27%	75	28%
Fetal death	6	9%	22	11%	28	11%
TOP	40	60%	123	62%	163	61%

*T21* trisomy 21, *T18* trisomy 18, *T13* trisomy 13, *PNS1* prenatal screening in first trimester, *PNS2* prenatal screening in second trimester, *PND* prenatal diagnosis, *TOP* termination of pregnancy

*7 cases with missing maternal age: all came from period 2007–2012, 1 case performed PNS1 and 6 had no known PNS or PND, all had a postnatal diagnosis of T21 and were live births

**p < 0.05

**Table 3:** First prenatal test, time of diagnosis and pregnancy outcome according to year of birth in cases with a common trisomy (T21, T18, T13) in cases with T21 and cases with T13/18

	Year of birth					
	2005–2006		2007–2012		Total	
Trisomy 21	*N* = 81		*N* = 250		*N* = 331	
First prenatal test*						
PNS1	28	35%	93	37%	121	37%
PNS2	2	2%	82	33%	84	25%
PND	11	14%	16	6%	27	27%
No PNS or PND	40	49%	59	24%	99	30%
Time of diagnosis						
Postnatal	44	54%	134	54%	178	54%
Prenatal < 24 weeks	35	43%	107	43%	142	43%
Prenatal > = 24 weeks	2	2%	9	4%	11	3%
Pregnancy outcome						
Live birth	46	57%	139	56%	185	56%
Fetal death	2	2%	20	8%	22	7%
TOP	33	41%	91	36%	124	37%
Trisomy 13 and 18	*N* = 47		*N* = 125		*N* = 172	
First prenatal test*						
PNS1	19	40%	66	53%	85	49%
PNS2	2	4%	27	22%	29	17%
PND	18	38%	21	17%	39	23%
No PNS or PND	8	17%	11	9%	19	11%
Time of diagnosis*						
Postnatal	8	17%	16	13%	24	14%
Prenatal < 24 weeks	29	62%	105	84%	134	78%
Prenatal > = 24 weeks	10	21%	4	3%	14	8%
Pregnancy outcome						
Live birth	9	19%	16	13%	25	15%
Fetal death	12	26%	19	15%	31	18%
TOP	26	55%	90	72%	116	67%

*T21* trisomy 21, *T18* trisomy 18, *T13* trisomy 13, *PNS1* prenatal screening in first trimester, *PNS2* prenatal screening in second trimester, *PND* prenatal diagnosis, *TOP* termination of pregnancy

*p < 0.05

### Prenatal tests performed

Before implementation 62% of the cases had a PNS1, PNS2 or PND performed, while after implementation the proportion increased to 79% in 2007–2009 and 84% in 2010–2012, see Table 1. This increase is mainly due to the higher uptake of PNS2 after the implementation, while the uptake of PND without prior screening tests decreased (*p* < 0.01).

**Fig. 1:**
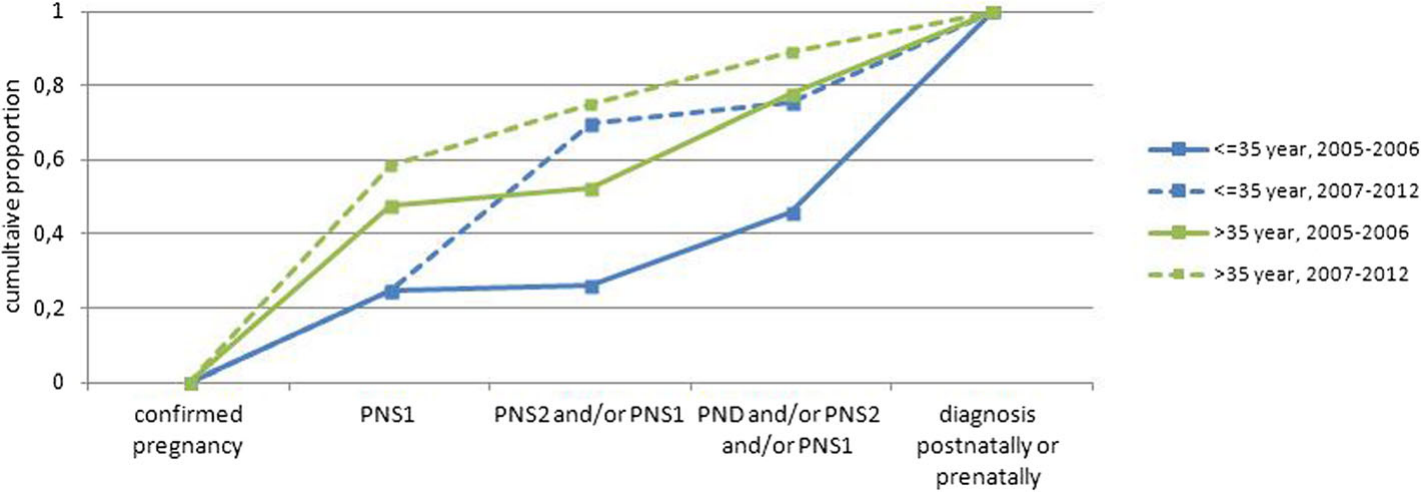
First prenatal test performed in cases with a common trisomy according to year of birth and maternal age. Abbreviations: PNS1 prenatal screening in first trimester, PNS2 prenatal screening in second trimester, PND prenatal diagnosis

Table 2 and Fig. 1 also point to difference between the age groups. In general, PNS1 was more frequently performed in women >35 years, than in women < =35 years: 56% versus 25%. This difference was observed before and after implementation, but with an increase in PNS1 performed in women >35 year from 54% in 2007–2009 to 62% in 2010–2012. After the implementation, the proportion of women < =35 years that had a screening test performed in their pregnancy (PNS1, PNS2 or both) was 70, and 75% of the women >35 years. Prenatal diagnosis without prior screening decreased in the group women < =35 year from 20% before to 5% after implementation and in the group women over 35 years and older from 25 to 14% (Table 2).

### Time of diagnosis

In 60% of the total cohort the diagnosis was made prenatally. This percentage did not change over time, although the proportion of cases that was diagnosed <24 weeks increased from 50% before to 57% after implementation (Table 1, (*p* = 0.054). Overall, in the age group < =35 years the diagnosis was made prenatally in 47% of cases, while in the age group >35 years the diagnosis was made prenatally in 73% of cases (*p* < 0.01). Although, in both maternal age groups the proportion of cases diagnosed postnatally remained similar after the implementation, there was a shift in timing of the prenatally diagnosed cases in the group < =35 year. In this age group, 30% were detected before 24 weeks of gestation before implementation; after implementation this proportion increased to 43% (*p* = 0.09, Table 2). T21 was less frequently diagnosed prenatally than T13/T18, but no shift occurred in time of diagnosis of T21 (*p* = 0.885, Table 3). However, for the T13/T18 cases the diagnosis was made earlier with more cases detected before 24 weeks of gestation 62% before versus 84% after implementation (*p* < 0.01, Table 3). The majority of the diagnosis in T13/T18 cases was made before 18 weeks gestation, but the proportion diagnosed before 18 weeks did not increase in 2010–2012 (54%) compared to 2005–2006 (49%) and 2007–2009 (61%). There was an increase in the proportion diagnosed between 18 and 23 weeks, from 13% in 2005–2006 to 30% in 2010–2012.

### Pregnancy outcome

After implementation of the PNS no significant shift occurred in pregnancy outcome (*p* = 0.913) compared to the period before implantation in the entire cohort (Table 1). Termination of pregnancy was performed in 87% of the prenatally diagnosed cases.

No significant shift occurred in pregnancy outcome in both maternal age groups since the implementation of PNS. But, overall, in the group < =35 year more children with trisomies were live born: 56% versus 28% in women >35 years, whereas in the group >35 year more pregnancies were terminated: 61% compared to 33%, Table 2 (*p* < 0.01).

In the T13/T18 group the percentage of fetal deaths decreased since the implementation from 26 to 15% and the percentage of TOPs increased from 55 to 72% (Table 3). This shift in pregnancy outcomes was however not statistically significant (*p* = 0.112).

## Discussion

In this population based study on prenatal detection of common trisomies after the introduction of a nationwide prenatal screening program, we found a shift in the proportion of women that had PNS performed, mainly due to an increase in the 20 weeks scan (PNS2). As a consequence more diagnoses were made earlier in pregnancy (<24 weeks) although the overall proportion of prenatally diagnosed cases remained at about 60% before and after implementation. However, there is a difference in time of diagnosis between both age groups: in women >35 years the diagnosis was made more often prenatally than in women < =35 years, which resulted in more TOPs among women of >35 years. The T13/T18 diagnosis is made earlier in pregnancy and in T13/T18 pregnancies fetal deaths decreased while TOPs increased.

The aim of the study was not to evaluate the performance of the PNS program but to evaluate the impact of the implementation of a nationwide program for PNS on the time of detection of common trisomies and subsequent pregnancy outcome. Although we could not cover the whole of the Netherlands in this study, we were able to include every recognized common trisomy case in the Northern Netherlands. The cytogenetic laboratory is the only laboratory in the region which performs these tests. Therefore we consider this study truly population based. Only cases, that were not karyotyped, could theoretically have been missed. We were not able to find out in every case if PNS was performed or not. Some cases would not have had performed PNS at all. Cases with only prenatal diagnosis for AMA and some fetal deaths could not have a PNS2 because the pregnancy was terminated or lost before the second trimester. Therefore both the uptake of PNS and PND were used in order to determine for which prenatal care the pregnant women opted first.

We included also PNS2 as a screening method for common trisomies. The detection of softmarkers and certain anomalies during PNS2 may indicate the presence of T21, T13 or T18, leading to an AC or CVS. In addition, PNS1 and PNS2 were both implemented as a part of the nation wide Dutch prenatal screening program at the same time in 2007 and therefore the influence of both is important leading to the detection of common trisomies. Before 2007, PNS was not carried out nation wide, but was nevertheless performed on individual basis. In the Northern Netherlands a second trimester serum screening has been offered on research basis since the 1990’s, but implementation was not supported nationwide by the Dutch Society of Obstetrics and Gynaecology [1]. Since the end of the 1990’s “scans for keepsake” were offered, without counselling about the possibility of the detection of fetal anomalies. The Dutch Health Council set the condition that quality of the counselling and the ultrasound performance should be guaranteed for SAS [12]. Therefore ultrasounds performed before 2007 were not considered as PNS2 in this study. Since the implementation, the quality of the SAS units is guaranteed and periodically audited by the regional screening centers which are orchestrated by the National Institute for Public Health and Environment. Information on PNS2 can be missing if there was only data from the laboratory available. Therefore the differences between the period before and the period after implementation might be less pronounced.

The uptake for PNS by pregnant women in the Northern Netherlands is estimated 17% for PNS1 risk assessment and 85% for PNS2 by SAS since the implementation [13]. Although the use standardized educational materials is mandatory in the counselling, the uptake for PNS1 in the Northern Netherlands is relatively low compared to the South-Western part of the Netherlands (28%) [14] and to other countries with uptakes reported between 48 and 97% [10, 15, 16]. These findings stress the importance of adequate counselling in which not only information is given about the conditions that are screened for and the tests that can be done, but also decision making support is provided by the counsellor to allow the woman to make an informed choice [17]. Different factors e.g., socio-economic status, attitude towards Downs’ syndrome and TOP, counselling, costs and knowledge about first trimester prenatal screening can influence the uptake. In the Northern Netherlands the main reason for the low uptake for PNS1 is the relatively positive attitude toward Downs’ syndrome and a negative attitude towards TOP [18]. Women of 35 years or younger tend to decline PNS1. The fact that the costs were not covered by the health insurance in this group may also have played a role. In our cohort the proportion of women that had PNS1 and/or PNS2 performed was similar in women < =35 and >35 years after the implementation of PNS. In other European countries several fetal scans are offered on a regular basis [19], while in the Netherlands SAS is offered after counselling and women are free to refrain from PNS2. If a SAS is not performed, as was the case before implementation, polyhydramnion, growth restriction or maternal symptoms in T18 and 13 becomes more evident in the 3^rd^ trimester.

No shift occurred in the time of detection (prenatally vs. postnatally) or pregnancy outcome since the implementation of prenatal screening, in contrast to many other countries where prenatal detection of common trisomies has increased and the live birth rate has decreased [2–6]. The relatively low uptake of PNS1 and the low prevalence of ultrasound anomalies in T21is the most likely explanation for the fact that the implementation of a nationwide PNS did not impact the prenatal detection and pregnancy outcome on a population level in the Northern Netherlands.

Although T13/T18 show more often ultrasound anomalies, still 14% was only detected postnatally in this study. A possible explanation could be that we considered diagnosis made after fetal deaths as postnatally diagnosed cases. Although no shift in prenatal or postnatal detection occurred, the implementation was accompanied with an increased proportion of cases diagnosed before 24 weeks. Detection before 24 weeks of pregnancy is important given the legal limit for TOP in the Netherlands of 24 weeks’ gestation. Interestingly, the main contributor to earlier diagnosis of T18 and T13 (<24 weeks) was the introduction of the routine 20 weeks scan, whose uptake is high.

Since April 2014, cell free (cf) DNA testing for T21, T13 and T18 has become available in the Netherlands as part of a national implementation research study: Trial by Dutch Laboratories for Evaluation of Non-Invasive Prenatal Testing (TRIDENT study) [20]. Women with an increased risk for trisomies (after a combined test, or because of a previous affected pregnancy) are offered this test. Since the introduction of cfDNA testing as second-tier test the number of invasive tests has decreased [21]. The availability of cfDNA testing as alternative to the CT, may lead to a higher uptake of prenatal screening [22] which may lead to a more pronounced effect on the outcome of pregnancies affected with a common trisomy than found in this study.

## Conclusion

This study showed that if the uptake of prenatal screening for chromosomal anomalies is low, the introduction of a national screening program has limited impact on the time of detection and outcome of the most common trisomies 21, 18 and 13. However, a low impact should not be mistaken for a low performance of the prenatal screening progam, as performance is measured by detection rate and false positive rate which we could not calculate with the available data. The uptake of the structural anomaly scan (PNS2) was much higher than of first trimester screening (PNS1). As a consequence more diagnoses were made < 24 weeks, but the overall proportion of prenatally diagnosed cases remained unchanged at about 60% before and after implementation. The only difference in time of diagnosis occurred in women >35 years, where the diagnosis was made more often prenatally leading to more TOP, than in women < =35 years. Owing to the overall higher rate of prenatally diagnosed T18 and 13 < 24 weeks, there was a shift from less fetal deaths to more TOPs.

## Abbreviations

AC: Amniocentesis
AFP: Alfa feto protein
AMA: Advanced maternal age
Cf DNA: Cell free DNA
CT: Combined test
CVS: Chorion villic sampling
EUROCAT: European network of population-based registries for the epidemiologic surveillance of congenital anomalies
fβ hCG: Free beta human chorionic gonadotropin
HCG: Human chorionic gonadotropin
MLPA: Multiplex ligation-dependent probe amplification
NT: Nuchal translucency
PAPP-A: Pregnancy associated plasma protein A
PND: Prenatal diagnosis
PNS: Prenatal screening
PNS1: Prenatal screening in first trimester
PNS2: Prenatal screening in second trimester
QF-PCR: Quantitative fluorescence-polymerase chain reaction
SAS: Structural anomaly scan
T13: Trisomy 13
T18: Trisomy 18
T21: Trisomy 21
TOP: Termination of pregnancy
TRIDENT: Trial by Dutch laboratories for evaluation of non-invasive prenatal testing
UMCG: University Medical Centre Groningen
*X*^2^: Chi square
